# Supplementary material for: The genome sequence of the Ruddy Highflyer, Hydriomena ruberata (Freyer, 1831) (Lepidoptera: Geometridae)
Source: Wellcome Open Res. 2026 Jun 13;11:353. [Version 1] doi: 10.12688/wellcomeopenres.26781.1 (PMC13639350; doi:10.12688/wellcomeopenres.26781.1)
Supplement: Peer review history

# Reviewer report: Reviewer response for version 1

**DOI:** 10.21956/wellcomeopenres.29487.r165544

**Published:** 2026-10-05

**Authors:** Xueyan Li

**Affiliations:** 1 Kunming Institute of Zoology, Kunming, Yunnan, China

In this MS, Dinca et al present a genome assembly from a male specimen of a moth *Hydriomena ruberata* (Geometridae). The data resource is very important for pest management and for investigating insect evolution. It can be indexed after some minor revisions.

**Minors:**

1. Background: when the species name “Hydriomena ruberata” first appeared, it was full spelling. After that, it can be spelt as “H. ruberata”.
2. Figure 1: please add the scale bar.

Are sufficient details of methods and materials provided to allow replication by others?

Yes

Is the rationale for creating the dataset(s) clearly described?

Yes

Are the datasets clearly presented in a useable and accessible format?

No

Are the protocols appropriate and is the work technically sound?

Yes

Reviewer Expertise:

entomology

I confirm that I have read this submission and believe that I have an appropriate level of expertise to confirm that it is of an acceptable scientific standard.

# Reviewer report: Reviewer response for version 1

**DOI:** 10.21956/wellcomeopenres.29487.r165540

**Published:** 2026-09-15

**Authors:** Scott D Cinel

**Affiliations:** 1 University of Florida, Gainesville, USA

This data note by Dinca *et al. *provides the first published genome assembly for the Ruddy Highflyer ( *Hydriomena ruberata*) as part of the Project Psyche initiative to generate genomes for all European lepidopteran species.  The text expertly details the specimen collection, tissue preparation, nucleic acid extraction, library preparation, and DNA sequencing technologies and all associated protocols used to generate this genome.  A main strength of this data note is the inclusion of both PacBio HiFi sequencing and Hi-C sequencing, which enabled chromosome-scale feature delineation and read mapping, and produced 2 separate haplotype-resolved genomes. Further, the assembled genomes have been made readily available in online repositories, and all accession information is clearly presented. Finally, all genome assembly curation and quality assessment procedures undergone by the authors are documented with precise detail, providing a readily reproducible workflow for future sequencing projects on closely related lepidopteran species.

 I especially appreciate the inclusion of painted Merian elements shown along each chromosome, as these colorful figures provide interesting detail regarding the arrangement of genes derived from ancestral linkage groups both within a single chromosome and on separate chromosomes, in some cases.

 Overall, this data note is of very high quality and, apart from generating a reference genome for the Ruddy Highflyer moth, also provides a wealth of valuable information and guidance for future studies on other species in the *Hydriomena* genus and those focusing more closely on the biology and ecology of *Hydriomena ruberata* specifically.

 The following are small typographical issues:

 In the PowerPoint slide version of Figure 4, the final word of the figure caption "ruberata" is missing from the text positioned above the figure.

 It appears that every time a figure is cited in parentheses, there is an unneeded space between the opening parenthesis and the letter "F."

 In the Hi-C section titled "Sample preparation and crosslinking," it is unclear what "TC" stands for in "TC buffer."

 In the "Assembly quality assessment section," the program name "Merqury.FK" should be changed to "MerquryFK."

 In the "Assembly quality metrics" section, the sentence " Table 4 lists the assembly metric benchmarks adapted from Rhie *et al.* (2021) the Earth BioGenome Project Report on Assembly Standards January 2026" can be improved by adding "and the" immediately after citing Rhie *et al.* (2021). 

 In the "Author information" section, as of this writing all links to zenodo.org are inoperable, but this is likely due to an issue on Zenodo's side and may fix itself with time.

Are sufficient details of methods and materials provided to allow replication by others?

Yes

Is the rationale for creating the dataset(s) clearly described?

Yes

Are the datasets clearly presented in a useable and accessible format?

Yes

Are the protocols appropriate and is the work technically sound?

Yes

Reviewer Expertise:

My background is in insect ecology and evolution broadly, with specific expertise in lepidopteran genomics and transcriptomics.

I confirm that I have read this submission and believe that I have an appropriate level of expertise to confirm that it is of an acceptable scientific standard.
